# Prevalence and Clinical Patterns of Piriformis Syndrome Among Actively Competing and Retired Elite Hockey Players

**DOI:** 10.3390/sports14030095

**Published:** 2026-03-03

**Authors:** Caleb Neal, Timothy Gelatt, Milan Toma

**Affiliations:** 1 Department of Osteopathic Manipulative Medicine, College of Osteopathic Medicine, New York Institute of Technology, Old Westbury, NY 11568, USA; caleb.w.neal@gmail.com; 2 Department of Emergency Medicine, North Shore University Hospital, Northwell Health 300 Community Drive, Manhasset, NY 11030, USA; 3 Rowan-Virtua School of Osteopathic Medicine, Rowan University, Stratford, NJ 08084, USA; gelatt75@rowan.edu

**Keywords:** piriformis syndrome, ice hockey, prevalence, athletic injuries, musculoskeletal disorders

## Abstract

Piriformis syndrome, a neuromuscular disorder caused by sciatic nerve compression by the piriformis muscle, remains understudied in athletic populations despite anecdotal reports of elevated prevalence in hockey players. This study investigated the prevalence of piriformis syndrome symptoms and potential risk factors in actively competing (current) and retired (former) high-level hockey players. A cross-sectional survey was conducted among 67 actively competing and retired professional, collegiate, and junior hockey players (58 males, 9 females; mean age 25.6 ± 4.0 years; mean playing experience 17.8 ± 3.7 years). Active playing status was defined as currently participating in organized competitive hockey at any level, while retired status was defined as having ceased competitive participation for at least one season. The survey instrument was based on a validated clinical assessment scoring system, consisting of 12 questions assessing piriformis syndrome-related symptoms. Participants were classified as “high score” (≥6 affirmative responses) or “low score” (<6 responses). Multiple linear regression analysis was used to evaluate associations between demographic variables (age, playing status, years played, competitive level) and total symptom scores. Overall, 25.4% (*n* = 17) of participants met criteria for high symptom burden, with sitting-induced buttock pain being the most prevalent specific symptom (40.3%). Mean total score was 4.8 ± 1.8 (range 2–10). Multiple regression analysis revealed no statistically significant associations between piriformis syndrome scores and any demographic variable (overall model: $R^{2}$ = 0.065, *p* = 0.374). Retired players showed a non-significant trend toward higher scores compared to actively competing players (β = −1.388, 95% CI: −2.793 to 0.018, *p* = 0.053). No correlations were observed with age (*r* = −0.045, *p* = 0.719), years played (*r* = −0.054, *p* = 0.666), or competitive level (*p* = 0.666). In conclusion, this study revealed substantial piriformis syndrome symptom burden (25.4%) in high-level hockey players without significant demographic associations.

## 1. Introduction

Piriformis syndrome is a neuromuscular disorder caused by sciatic nerve compression by the piriformis muscle, producing sciatica-like symptoms [1,2,3,4]. Prevalence among low back pain patients ranges from 5% to 36% [5], with 6% incidence in patients with similar symptoms [6]. Anatomical variations account for up to 16.2% of surgical cases [7], though the syndrome remains underdiagnosed [4,6].

While hockey players are prone to numerous MSK injuries [8,9,10,11], including training-related injury patterns documented in field hockey populations [12] and musculoskeletal concerns affecting female athletes across hockey variants [13], specific piriformis syndrome rates in former college and professional players remain uninvestigated, representing a literature gap warranting further research.

### 1.1. Clinical Definition and Classification

The terminology and classification of piriformis syndrome remains controversial in the modern literature [14]. While this study employs the Michel et al. scoring system referencing piriformis syndrome [15], some authors advocate for the broader classification of Deep Gluteal Syndrome [16,17,18], which encompasses sciatic nerve entrapment by various anatomical structures beyond the piriformis muscle alone, including the obturator internus, gemelli muscles, and fibrous bands [19]. The distinction between these diagnostic frameworks reflects ongoing debate regarding anatomical specificity versus clinical pragmatism in extra-spinal sciatic nerve compression [18,20].

### 1.2. Pathophysiology of Disease

Piriformis syndrome results from sciatic nerve compression by the piriformis muscle [11], with anatomical variations contributing to 16.2% of cases [21,22]. These MSK variations cause sensory, motor, and trophic dysfunctions [23], leading to chronic somatic dysfunction with pain, paresthesia, hypesthesia, and muscle weakness [24].

Contributing factors include anatomical variations, hypertrophy, trauma, and intramuscular masses [25,26,27]. Piriformis hypertrophy, observed in athletes including hockey players, increases sciatic nerve pressure [28]. Hip endoscopy advances have clarified pathophysiological mechanisms [21].

### 1.3. Diagnosis

Diagnosing piriformis syndrome is challenging due to overlapping symptoms with other low back and buttock pain conditions [11,29,30]. No gold standard clinical test exists [31], and consistent objective findings are lacking [32,33]. The condition is often misdiagnosed as lumbar discopathy [34] and complicated by rarity and nonspecific symptoms [35].

Diagnosis relies on medical history, physical examination, and diagnostic tests [36], with electrophysiologic evaluation historically considered the mainstay [37]. Modalities include EMG, CT, MRI, bone scan [2,38], ultrasound, electrophysiologic studies, and scintigraphy [39].

### 1.4. Treatment

Conservative treatments include physical therapy, lifestyle modification, NSAIDs, muscle relaxants, and neuropathic pain medications [18,40,41], with OMT used alone or combined with pharmacotherapy [29,42]. Techniques such as myofascial release, hip abductor strengthening, and integrated neuromuscular inhibition show effectiveness [18,43,44].

Interventional options include piriformis injections with local anesthetic/steroids or BoTox [45,46], ultrasound-guided injection with EMG confirmation [47], PrP injection [48], sacral nerve stimulation, and sacroiliac joint manipulation [49,50].

Surgical intervention, reserved for severe refractory cases, includes laparoscopic intrapelvic sciatic nerve decompression and piriformis resection [51,52,53,54,55]. Recent systematic reviews have evaluated outcomes and complications of both open and endoscopic surgical approaches [54,55], though recurrence may occur [56].

Recent systematic reviews synthesizing evidence on piriformis syndrome and deep gluteal syndrome management have evaluated conservative treatment modalities [18], surgical outcomes comparing endoscopic and open techniques [54], and endoscopic approaches specifically for sciatic nerve entrapment [55], providing detailed modern evidence synthesis for clinical decision-making [4].

### 1.5. Analysis of Gaps in Literature

Critical knowledge gaps exist requiring investigation. Comprehensive studies are needed on precise etiology and risk factors to develop targeted prevention and treatment strategies. Comparative effectiveness studies evaluating different treatment modalities’ efficacy, safety, and long-term outcomes would inform clinical decision-making.

Recent studies have begun characterizing sport-specific health profiles in hockey populations, including nutritional status and anthropometric profiles in ice hockey players [13], injury patterns in field hockey athletes [12], and performance parameters in rink hockey players [57,58]. However, knowledge gaps persist regarding sleep disturbances and energy balance [59], and comprehensive musculoskeletal screening specific to piriformis syndrome remains absent across all hockey variants.

Novel diagnostic approaches, including advanced imaging and diagnostic algorithms, are needed given the absence of gold standard tests. Long-term follow-up studies assessing recurrence rates and treatment durability are essential. Patient-reported outcomes, quality-of-life measures, and functional assessments remain understudied.

Research on multidisciplinary management approaches involving physical therapy, pain management, neurology, and orthopedics collaboration warrants investigation. Addressing these gaps will facilitate evidence-based guideline development for diagnosis and management.

To address these knowledge gaps (namely, the complete absence of sport-specific musculoskeletal screening for piriformis syndrome in hockey athletes and the lack of systematic epidemiological data characterizing prevalence, symptom patterns, and associated risk factors) the present study was designed to provide the first systematic investigation of piriformis syndrome symptom burden in current and former high-level hockey players, thereby establishing the foundational epidemiological evidence necessary for development of targeted screening protocols and evidence-based clinical guidelines.

### 1.6. Study Objectives

This study aimed to: (1) establish the prevalence of piriformis syndrome symptoms in current and former high-level hockey players using a validated scoring system; (2) characterize symptom patterns and clinical presentations; (3) evaluate associations between symptom burden and demographic variables (age, sex, playing status, years of participation, competitive level); and (4) assess whether cumulative athletic exposure demonstrates a dose–response relationship with symptom severity. These findings aim to inform evidence-based screening and management strategies for this at-risk athletic population.

## 2. Methods

### 2.1. Study Design

This cross-sectional survey investigated piriformis syndrome prevalence in current and former high-level hockey players. Surveys were administered electronically via Research Electronic Data Capture (REDCap) between May and November 2024.

### 2.2. Sample

Current and former professional, collegiate, and junior hockey players were recruited via telephone and social media. Of 75 athletes agreeing to participate, 67 completed the full survey (89.3% completion rate). Eight were excluded: six for incomplete responses, two for data quality concerns.

### 2.3. Variables

The survey was based on the validated clinical assessment by Michel et al. [15], consisting of 12 questions assessing piriformis syndrome-related symptoms: (Q1) buttock pain with diurnal variation; (Q2) chronic lower back pain; (Q3) spinal tenderness (L2–S1); (Q4) straight leg raise equivalence; (Q5) prolonged sitting-induced symptoms; (Q6) sciatic distribution pain; (Q7) pain with FAIR/Freiberg/HCLK maneuvers; (Q8) isometric exercise-induced pain; (Q9) palpation tenderness over piriformis; (Q10) L5–S1 pain with sustained stretching; (Q11) L5–S1 pain with sustained resistance exercises; (Q12) perineal pain.

It should be noted that the Michel et al. scoring system was originally developed for in-person clinical assessment rather than self-reported surveys [15]. Several items, including FAIR/Freiberg/HCLK maneuvers (Q7), palpation tenderness over piriformis (Q9), and spinal tenderness assessment (Q3), are inherently clinician-dependent and may not be reliably self-assessed by participants without clinical demonstration. Consequently, this study assesses piriformis syndrome symptom burden rather than definitive clinical diagnosis, a distinction maintained throughout the manuscript.

Participants provided age, sex, playing status (current/former), years of organized hockey experience (excluding recreational play), and highest competitive level: (1) Junior, (2) Collegiate (NCAA, ACHA, AUA), or (3) Professional (FHL, SPHL, European leagues).

### 2.4. Procedure

Questions were scored dichotomously following Michel et al. [15]. Questions 1, 5, 6, 7, 8, 10, and 11 scored 1 for affirmative responses; Questions 2, 3, 4, 9, and 12 were reverse-coded (1 for negative responses). Composite scores ranged 0–12 points. Binary classification used threshold ≥6 points for “high score” (*n* = 17, 25.4%) versus <6 points for “low score” (*n* = 50, 74.6%).

Raw data were exported from REDCap to Microsoft Excel (Version 16.0). Playing status was coded as binary (current = 1, former = 0). Competitive level was converted to ordinal scale (Junior = 1, Collegiate = 2, Professional = 3).

### 2.5. Statistical Analysis

All statistical analyses were performed using custom MATLAB scripts (Version R2024a, The MathWorks, Inc., Natick, MA, USA) that imported data from Microsoft Excel spreadsheets. Data were read using the readtable function from the Statistics and Machine Learning Toolbox. Descriptive statistics were calculated using built-in MATLAB R2024a functions (mean, std, median, quantile). Simple and multiple linear regression analyses were performed using the fitlm function, which implements ordinary least squares estimation and provides comprehensive regression diagnostics including residual plots. Analysis of variance was conducted using the anova and anova1 functions. Correlation coefficients were computed using the corrcoef function. MATLAB was selected for this study due to: (1) robust statistical capabilities with well-documented, peer-reviewed algorithms; (2) modular script architecture allowing transparent inspection of all analytical steps and independent verification; and (3) adequacy for the analytical methods employed (descriptive statistics, simple and multiple linear regression, ANOVA), which are implemented using standard statistical procedures.

Continuous variables (age, years of experience, total score) were summarized using means, standard deviations, medians, interquartile ranges, and ranges. Categorical variables (sex, playing status, competitive level) used frequencies and percentages. Distributions were visualized using bar charts and histograms. Significance threshold: α=0.05.

Relationships between predictors and scores were assessed using simple linear regression for continuous variables (age, years played) and ANOVA for categorical variables (playing status, competitive level). Scatter plots with fitted regression lines and Pearson correlation coefficients (*r*) quantified linear relationships. Box plots visualized score distributions across competitive levels.

Multiple regression evaluated simultaneous effects of demographic variables:

(1) $$\mathrm{Score}=\beta_{0}+\beta_{1}\left(\mathrm{Age}\right)+\beta_{2}\left(\mathrm{Playing}\;\mathrm{Status}\right)+\beta_{3}\left(\mathrm{Years}\;\mathrm{Played}\right)+\beta_{4}\left(\mathrm{Level}\right)+\epsilon ,$$

where Score is the dependent variable, $\beta_{0}$ is the intercept, $\beta_{1}$–$\beta_{4}$ are regression coefficients, and ϵ is the normally distributed error term. Prior to interpretation, multiple linear regression assumptions were evaluated and satisfied. Visual inspection of residual histograms and Q–Q plots indicated approximately normal distribution. Residual versus fitted value plots showed no systematic patterns suggesting heteroscedasticity. Variance inflation factors (VIF) were all <2.5, well below the threshold of concern (VIF < 10), indicating no problematic multicollinearity. Partial regression plots for continuous predictors supported linearity assumptions. Model fit was assessed using $R^{2}$, adjusted $R^{2}$, and overall F-statistic. Individual predictor significance used *t*-tests (α=0.05). The dichotomization of scores at the threshold of six affirmative responses, while consistent with the Michel et al. validation study [15], necessarily reduces statistical power compared to treating scores as continuous variables. This analytical choice was retained for clinical interpretability and comparability with the existing literature, though sensitivity analyses treating scores continuously (reported in the main regression model) were conducted to mitigate potential information loss. Results were visualized using forest plots with 95% confidence intervals.

Response rates were calculated as proportion of affirmative responses per question. Response patterns across demographic subgroups were examined using heatmap visualization for five questions with intermediate prevalence (Q1, Q5, Q6, Q10, Q11), calculated separately for playing status, age groups (18–23, 24–27, 28–33), sex, and competitive level (junior excluded due to small sample).

All visualizations used TikZ and PGFPlots in L^A^T_E_X (Version 3.1.10). Two-tailed tests with α=0.05 were used for all hypothesis testing. Raw data and analysis code are available upon reasonable request.

#### Sample Size and Power Considerations

No a priori sample size calculation was performed prior to data collection. Post hoc power analysis was conducted to evaluate the adequacy of the achieved sample size (*n* = 67) for detecting associations between demographic variables and piriformis syndrome symptom burden. For the multiple linear regression analysis with four predictors (age, playing status, years played, competitive level) and the observed effect size ($R^{2}$ = 0.065, $f^{2}$ = 0.0695), the achieved statistical power was approximately 30–35% at α = 0.05. To achieve the conventional 80% power threshold for detecting an effect of this magnitude, approximately 120–130 participants would be required [60].

For the prevalence estimate of high piriformis syndrome symptom scores (25.4%), the 95% confidence interval ranged from 15.0% to 35.8%, corresponding to a margin of error of ±10.4%. To achieve a more precise prevalence estimate with a ±5% margin of error, approximately 290 participants would be required. The near-significant association observed for playing status (*p* = 0.053, effect size d≈ 0.79) suggests the current sample provided approximately 75–80% power to detect this medium-to-large effect, indicating that the borderline result may reflect genuine biological signal rather than insufficient statistical power. These power limitations should be considered when interpreting null findings for other predictors and underscore the need for replication studies with larger samples to definitively characterize risk factor associations in this population.

## 3. Results

**Figure 1:**
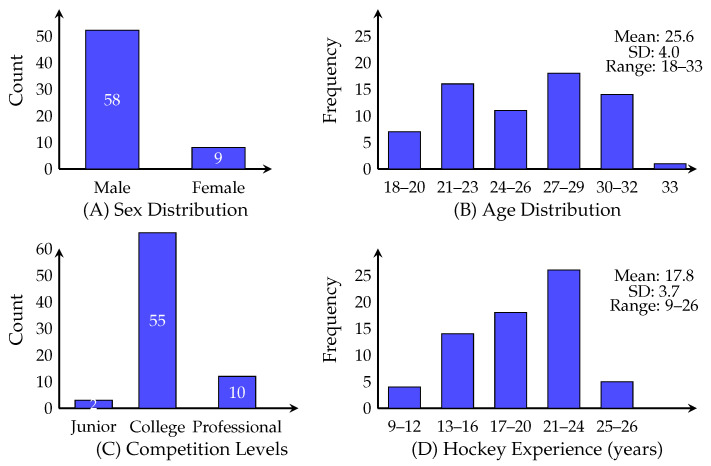
Participant demographics and hockey experience distribution. Panel (**A**) shows the sex distribution with 58 males (86.6%) and 9 females (13.4%). Panel (**B**) displays the age distribution histogram showing a mean age of 25.6 ± 4.0 years with a range from 18 to 33 years. Panel (**C**) presents the distribution of highest competitive levels achieved, with 2 participants (3.0%) having played only junior hockey, 55 participants (82.1%) reaching collegiate level, and 10 participants (14.9%) achieving professional status. Panel (**D**) illustrates the distribution of years of organized hockey experience, with a mean of 17.8 ± 3.7 years and a range from 9 to 26 years of experience.

**Figure 2:**
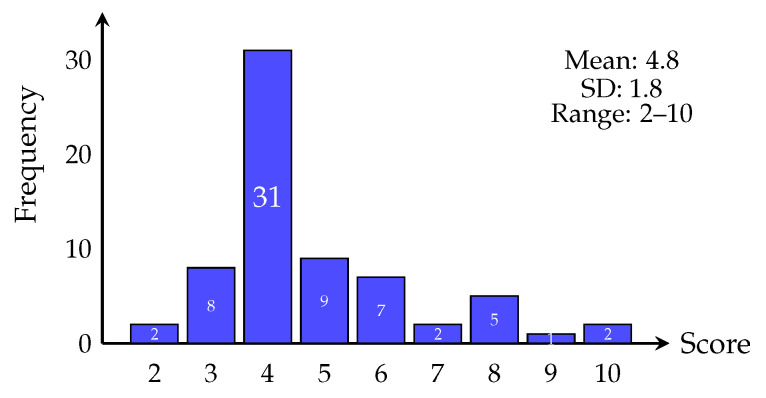
Distribution of total piriformis syndrome scores across all participants (*n* = 67). The histogram shows a right-skewed distribution with scores ranging from 2 to 10, a mean of 4.8 ± 1.8, and a modal score of 4 (46.3% of participants, *n* = 31). The distribution indicates that most participants experienced relatively low symptom burden, with only 14.9% (*n* = 10) scoring at or above the clinical threshold of 6 points. The right tail of the distribution extends to a maximum score of 10, suggesting a subgroup of participants with substantially elevated symptom burden.

**Figure 3:**
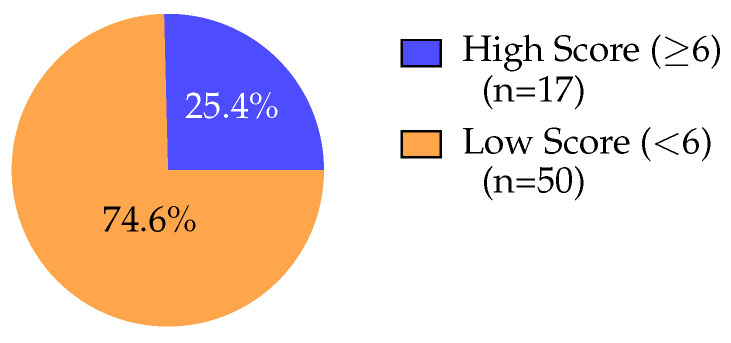
Binary outcome classification based on the clinical threshold of six or more affirmative responses. The pie chart reveals that 17 participants (25.4%) were classified as “high score,” meeting the Michel et al. criteria for elevated piriformis syndrome symptom burden, while 50 participants (74.6%) were classified as “low score.” This binary classification provides a clinically interpretable prevalence estimate, indicating that approximately one-quarter of current and former high-level hockey players in this sample demonstrated symptom profiles consistent with piriformis syndrome. The relatively high prevalence of elevated symptom burden warrants consideration of systematic screening protocols in this athletic population.

**Figure 4:**
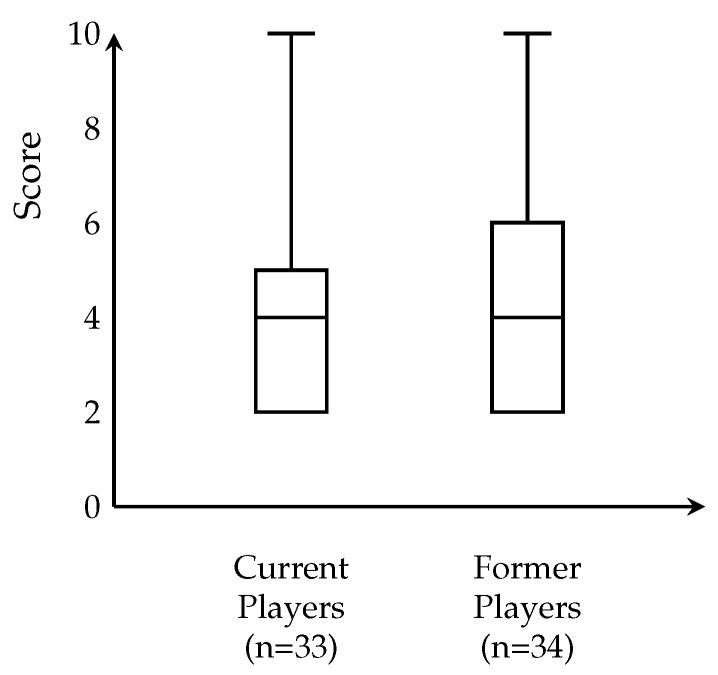
Box plot comparison of piriformis syndrome scores between current players (*n* = 33) and former players (*n* = 34). Both groups demonstrated identical median scores of 4 points, though the distributions differed slightly in spread. Current players displayed a narrower interquartile range (IQR: 4–5) compared to former players (IQR: 4–6), suggesting greater homogeneity in symptom burden among actively competing athletes. Both groups shared identical minimum (2) and maximum (10) values, indicating comparable ranges of symptom severity. The difference in score distributions approached but did not reach statistical significance (*p* = 0.053), representing a near-significant trend toward higher symptom burden in former players that warrants further investigation in larger cohorts and longitudinal studies.

**Figure 5:**
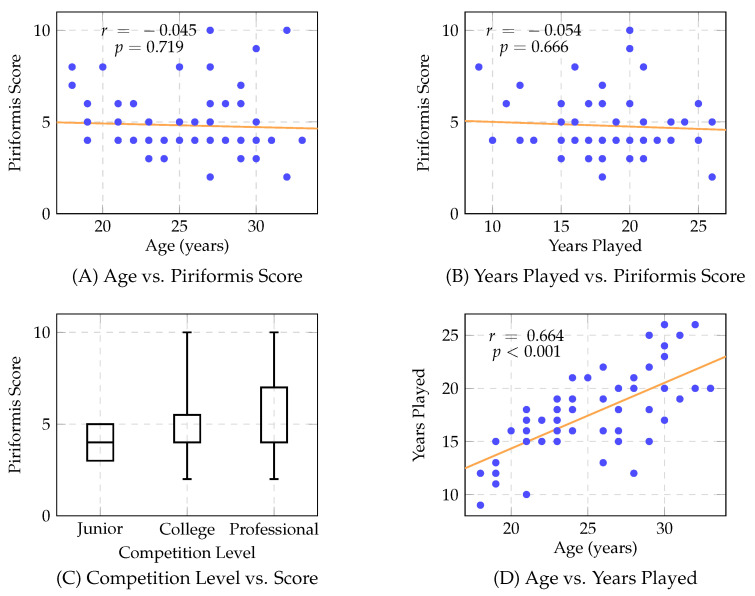
Correlation analysis between hockey career variables and piriformis syndrome scores. Panel (**A**) shows a scatter plot of age versus piriformis score with regression line, revealing no significant correlation (r=−0.045, p=0.719). Panel (**B**) displays a scatter plot of years played versus piriformis score with regression line, also showing no significant association (r=−0.054, p=0.666). Panel (**C**) presents box plots comparing piriformis scores across three competitive levels (Junior: n=2, College: n=55, Professional: n=10), demonstrating similar score distributions across levels with no significant differences (p=0.666). Panel (**D**) illustrates a scatter plot of age versus years played, revealing a strong positive correlation as expected (r=0.664, p<0.001), validating the internal consistency of the dataset.

**Figure 6:**
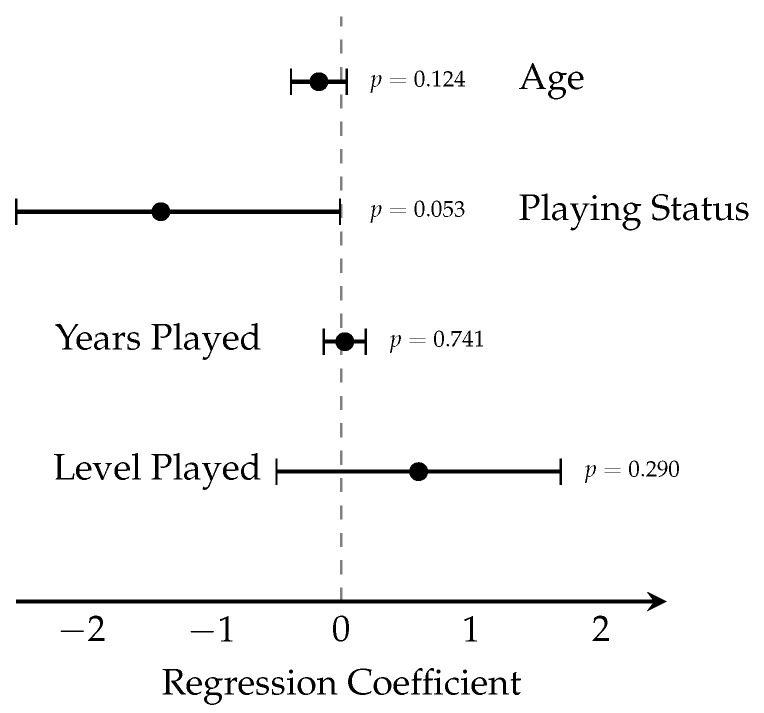
Multiple regression analysis forest plot showing regression coefficients and 95% confidence intervals for all predictor variables (*n* = 67). No statistically significant associations were observed between any demographic variable and piriformis syndrome scores. The dashed vertical line at zero represents the null hypothesis of no effect. The overall regression model was not significant ($F_{4,62}=1.08$, p=0.374, $R^{2}=0.065$).

**Figure 7:**
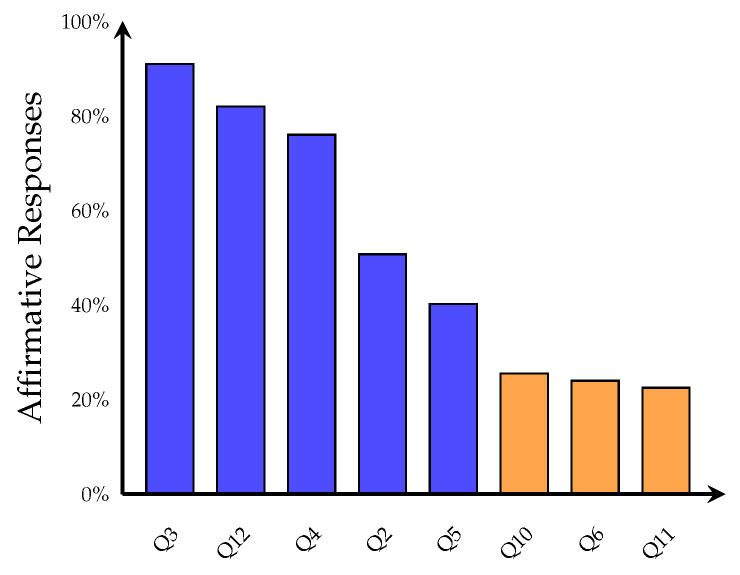
Prevalence of affirmative responses for individual survey questions (*n* = 67), ranked by frequency. Questions are based on the Michel et al. clinical assessment system. The three highest-prevalence items were absence of spinal tenderness (Q3, 91.0%), absence of perineal pain (Q12, 82.1%), and equivalent straight leg raise test (Q4, 76.1%). Among classic piriformis syndrome symptoms, sitting-induced buttock pain (Q5) showed the highest prevalence at 40.3%, followed by pain with sustained L5–S1 stretching (Q10, 25.4%), sciatic distribution pain (Q6, 23.9%), and pain with resistance exercises (Q11, 22.4%). Questions are color-coded to distinguish between general assessment items/negative findings (blue) and classic piriformis syndrome-specific symptoms (orange).

**Figure 8:**
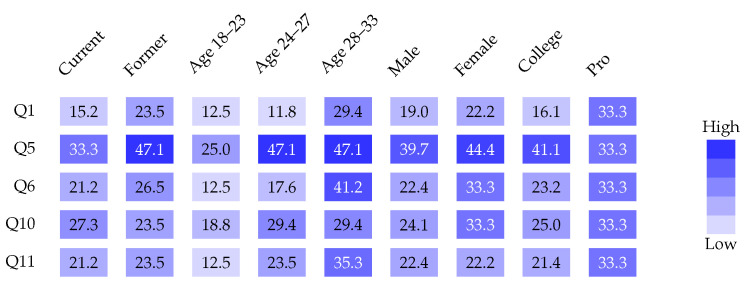
Heatmap of response patterns across demographic subgroups for selected questions with intermediate prevalence (Q1, Q5, Q6, Q10, Q11). Each cell displays the percentage of affirmative responses within that specific demographic category, with color intensity proportional to response rate (lighter blue indicating lower percentages, darker blue indicating higher percentages). The analysis demonstrates relatively homogeneous symptom distributions across most demographic categories, with slight variations by age group and playing status. The 28–33 age group showed elevated rates ranging from 29.4% to 47.1%, and former players demonstrated response rates of 23.5% to 47.1% across the selected questions. Junior-level players were excluded from this analysis due to small sample size (*n* = 2).

Results characterize demographic composition (Figure 1), score distribution (Figure 2), binary outcome classification (Figure 3), playing status comparison (Figure 4), bivariate correlations (Figure 5), multiple regression analysis (Figure 6), individual question response patterns (Figure 7), and demographic response patterns (Figure 8). Statistical significance was evaluated at α=0.05 with 95% confidence intervals.

Demographic characteristics are illustrated in Figure 1 and summarized in Table 1. The sample was predominantly male (86.6%, *n* = 58) with female representation (13.4%, *n* = 9). Age distribution showed the largest representation in 27–29 years (*n* = 18, 26.9%) and 30–32 years (*n* = 16, 23.9%). Competitive level was heavily collegiate (*n* = 55, 82.1%), with junior-only (*n* = 2, 3.0%) and professional (*n* = 10, 14.9%) players. Hockey experience ranged 9–26 years, with modal category 21–24 years (*n* = 26, 38.8%), and 73.1% (*n* = 49) playing ≥17 years.

Score distribution (Figure 2, Table 2) was right-skewed, with 46.3% (*n* = 31) scoring 4 points. Only 14.9% (*n* = 10) scored ≥6. Binary classification (Figure 3, Table 2) revealed that 17 participants (25.4%) met high symptom burden criteria versus 50 (74.6%) low burden. Current versus former players (Figure 4, Table 2) showed similar distributions (median = 4), with current players displaying narrower IQR (4–5) versus former players (4–6), not reaching significance (*p* = 0.053).

Correlation analyses (Figure 5) revealed no significant associations. Age showed no correlation with scores (r=−0.045, F=0.131, p=0.719) (Figure 5A). Years played showed no correlation (r=−0.054, F=0.188, p=0.666) (Figure 5B). Competitive level analysis (Figure 5C) revealed comparable distributions, namely, junior (*n* = 2, median = 4, range: 3–5), collegiate (*n* = 55, median = 4, IQR: 4–5.5, range: 2–10), and professional (*n* = 10, median = 4, IQR: 4–7, range: 2–10), with no significant relationship (F=0.188, p=0.666). Age and years played demonstrated an expected strong correlation (r=0.664, p<0.001) (Figure 5D), validating data reliability.

Multiple linear regression (Figure 6) was not significant ($F_{4,62}=1.08$, p=0.374, $R^{2}=0.065$). No individual predictors showed significance: age (β=−0.172, 95% CI: −0.392 to 0.048, p=0.124), playing status (β=−1.388, 95% CI: −2.793 to 0.018, p=0.053), years played (β=0.027, 95% CI: −0.136 to 0.191, p=0.741), or competitive level (β=0.595, 95% CI: −0.520 to 1.710, p=0.290).

Individual question responses (Figure 7) showed the highest affirmative rates for absence of spinal tenderness (Q3, 91.0%) and absence of perineal pain (Q12, 82.1%). Classic symptoms showed moderate prevalence: sitting-induced pain (Q5, 40.3%), pain with sustained L5–S1 stretching (Q10, 25.4%), FAIR/Freiberg/HCLK maneuver pain (Q6, 23.9%), resistance exercise pain (Q11, 22.4%), and buttock pain with diurnal variation (Q1, 19.4%). Chronic lower back pain was reported by 49.3% (reverse-coded Q2). Response patterns across demographic subgroups (Figure 8) revealed homogeneous distributions with no distinct clustering.

## 4. Discussion

This study systematically investigated piriformis syndrome prevalence in 67 current and former high-level hockey players. While 25.4% demonstrated high symptom scores and 40.3% reported sitting-induced buttock pain, demographic and career variables explained only 6.5% of score variance with no individual predictor achieving significance. The near-significant former player trend (p=0.053) and absence of correlation with years played (p=0.741) challenge conventional cumulative-exposure models.

### 4.1. Interpretation of Null Career-Related Associations

#### 4.1.1. Anatomical Predisposition Hypothesis

The absence of significant associations between piriformis syndrome scores and cumulative exposure variables (years played, age) is consistent with, though does not definitively establish, anatomical predisposition as a primary determinant. Published anatomical studies report that approximately 15–20% of cadaveric specimens demonstrate sciatic nerve anatomical variants, including nerve passage through rather than below the piriformis muscle, bifid muscle morphology, and aberrant nerve pathways [21,22]. These congenital variations, if present in our cohort, would not be expected to correlate with playing duration. However, our study did not include anatomical imaging to directly assess nerve or muscle variants, limiting this interpretation to plausible hypothesis rather than demonstrated mechanism. The skating motion’s repetitive external rotation and hip flexion may unmask pre-existing anatomical vulnerability rather than create acquired pathology, functioning as a biomechanical stress test for underlying structural predisposition.

#### 4.1.2. Early Saturation and Plateau Model

Alternatively, symptoms may develop early then plateau rather than progress linearly. Protective neuromuscular adaptations (compensatory synergist activation, altered movement patterns, strengthened supporting musculature) may counterbalance mechanical stress, explaining why 74.6% score below clinical threshold. The strong age–years played correlation (r=0.664, p<0.001) validates data quality, confirming that null piriformis findings reflect genuine biology rather than measurement error.

#### 4.1.3. Individual Susceptibility over Population Risk

Symptom pattern homogeneity across demographic subgroups (Figure 8) supports individual factors overwhelming population-level patterns, contrasting sharply with conditions like chronic traumatic encephalopathy or osteoarthritis showing clear dose–response relationships.

### 4.2. Prevalence and Clinical Significance

The 25.4% prevalence represents an intermediate value between general population estimates (5–6%) and low back pain patients (up to 36%), potentially reflecting: (1) symptom assessment versus confirmed diagnoses, (2) genuine hockey-specific risk, or (3) subclinical disease burden. Low mean scores (4.8 ± 1.8) with right-skewed distribution suggest most participants experience routine strain rather than pathological compression.

The 40.3% sitting pain prevalence, nearly double the overall high-score rate, suggests either a sensitive but non-specific early marker, adaptive changes to skating biomechanics (piriformis hypertrophy producing discomfort without true syndrome), or misattribution to alternative diagnoses (ischial bursitis, hamstring tendinopathy, gluteal strain). Regardless, this warrants systematic querying during preparticipation examinations and post-career assessments.

### 4.3. The Former Player Trend: Interpretation of p=0.053

The near-significant 1.4-point elevation in former players may reflect: (1) decompensation following training cessation unmasking pre-existing dysfunction, (2) cumulative microtrauma with delayed manifestation (though lack of correlation with years played argues against simple linear accumulation), or (3) reporting bias with current players underreporting due to pain normalization or competitive pressure. Post hoc power analysis indicates the current sample provided approximately 75–80% power to detect this medium-to-large effect size (d≈0.79), suggesting that the borderline result (p=0.053, 95% CI: −2.793 to 0.018) may reflect a genuine biological signal approaching statistical significance rather than insufficient statistical power. Nevertheless, the confidence interval crossing zero and the exploratory nature of this finding mandate replication in larger cohorts before definitive conclusions.

### 4.4. Mechanistic Considerations and Biomechanical Context

Skating imposes sustained hip flexion, repetitive external rotation, and explosive push-off thousands of times per game, yet produces no linear risk accumulation; contrasting sharply with overhead-throwing athletes’ dose-dependent shoulder pathology, runners’ mileage-dependent stress fractures, and collision-sport athletes’ duration-dependent neurodegeneration.

Cross-sport comparison strengthens anatomical predisposition: ballet dancers and figure skaters report 6–45% prevalence; cyclists show elevated rates; while swimming and rowing show lower prevalence despite equivalent training volumes, supporting position-specific risk from anatomical stress patterns.

The absence of sport-specific comparative data for hockey limits interpretation. While recent research has characterized training-related injuries in field hockey [12], nutritional and anthropometric profiles in ice hockey [13], and biomechanical parameters in rink hockey [57,58], systematic musculoskeletal screening for piriformis syndrome has not been reported. Additionally, emerging evidence of sleep disturbances and energy imbalance in hockey populations [59] suggests broader health surveillance gaps that may obscure piriformis syndrome recognition and reporting.

These cross-sport comparisons, while hypothesis-generating, must be interpreted cautiously given methodological heterogeneity across studies, including varied diagnostic criteria, assessment methods, and population sampling. Direct comparative studies using standardized assessment protocols across multiple sports would be required to definitively establish relative risk profiles.

### 4.5. Implications for Clinical Practice and Screening

The absence of demographic predictors for symptom burden, combined with substantial overall prevalence, suggests that if screening is to be implemented, universal rather than risk-stratified approaches may be warranted. However, several important qualifications must be emphasized. First, our findings are preliminary and exploratory, derived from a cross-sectional survey without clinical confirmation. Universal screening in any population requires demonstration of cost-effectiveness, diagnostic accuracy with acceptable sensitivity and specificity, and evidence that early detection leads to improved outcomes, none of which have been established for piriformis syndrome in hockey players. Second, any screening program should employ a two-stage approach: initial symptom-based questionnaire screening (maximizing sensitivity) followed by confirmatory clinical evaluation including physical examination maneuvers (FAIR, Freiberg, Beatty tests) and, where indicated, diagnostic imaging or injection (maximizing specificity). Survey-based screening alone is insufficient for diagnosis given the symptom overlap with alternative conditions including lumbar radiculopathy, sacroiliac joint dysfunction, and gluteal tendinopathy. Third, the clinical significance of identified cases remains unclear; the high prevalence of sitting-induced pain (40.3%) may reflect non-specific musculoskeletal discomfort rather than true syndrome requiring intervention. Therefore, our data support the consideration of systematic piriformis syndrome assessment as part of comprehensive musculoskeletal screening in hockey athletes, but implementation should be regarded as investigational pending validation studies demonstrating clinical utility and cost-effectiveness.

### 4.6. Differential Diagnosis and Diagnostic Validity

Lumbar radiculopathy, sacroiliac joint dysfunction, gluteal tendinopathy, and ischial bursitis overlap symptomatically [30]. The Michel system attempts discrimination through negative findings (91.0% lacked spinal tenderness) and positive piriformis-specific findings, but specificity in athletic populations remains unclear. Survey-based methodology introduces misclassification potential without in-person demonstration of examination maneuvers. Confirmatory studies incorporating physical examination, diagnostic injection, or MRI would clarify whether our prevalence represents symptom burden versus definitive diagnosis. A two-stage approach (namely, initial survey screening followed by confirmatory evaluation) balances efficiency with accuracy.

### 4.7. Study Limitations

Cross-sectional design precludes causal inference; longitudinal studies are needed. Survey methodology without clinical confirmation introduces misclassification bias, with the Michel system unvalidated for athletic cohorts or survey administration. Recall bias particularly affects former players. Absence of training intensity, practice volume, position, or injury history limits granular exposure assessment. Post hoc power analysis revealed that the achieved sample size (n=67) provided only 30–35% power to detect the observed overall effect size in the multiple regression model ($R^{2}=0.065$). Achieving 80% power for an effect of this magnitude would require approximately 120–130 participants. This limited statistical power increases Type II error risk for demographic predictors, meaning genuine small-to-moderate associations may have been missed. However, the playing status effect demonstrated adequate power (75–80%) despite borderline significance (p=0.053), and the consistency of null findings across multiple demographic variables suggests the absence of strong linear associations rather than systematic underpowering. The prevalence estimate (25.4%) carries substantial uncertainty (95% CI: 15.0–35.8%, margin of error ±10.4%), requiring approximately 290 participants to achieve a more precise estimate with ±5% margin of error.

Small subgroup samples (nine females, eight professionals, two juniors) severely limit power. Age restriction (18–33) excludes adolescents and long-retired players who might manifest delayed effects. Social media recruitment introduces selection bias. The sample composition limits generalizability in two important respects. First, female representation was limited (13.4%, *n* = 9), restricting sex-specific inferences and precluding definitive conclusions about piriformis syndrome patterns in female hockey players. Second, the age range (18–33 years, mean 25.6 ± 4.0) reflects current and recently retired athletes, excluding both adolescent players and long-retired individuals beyond early adulthood. Symptom patterns in these unrepresented groups, particularly delayed manifestations in athletes many years post-retirement, remain unknown. Absence of objective measures (examination, imaging, confirmatory testing) prevents definitively distinguishing piriformis syndrome from alternatives. Unmeasured confounders (BMI, concurrent injuries, other sports, occupation) and symptom severity/treatment data limit interpretation. Linear regression assumptions were untested; non-linear relationships and interactions unexplored. Modest $R^{2}$ (0.065) indicates that 93.5% variance remains unexplained.

These power limitations have important implications for interpretation. Null findings for age, years played, and competitive level cannot be definitively interpreted as absence of association; rather, they indicate that if associations exist, they are either very small or highly variable across individuals. The sample size was adequate to detect medium-to-large effects (as evidenced by the near-significant playing status finding) but insufficient to reliably detect small effects that may nonetheless be clinically meaningful at the population level.

### 4.8. Future Directions

Priorities include: prospective longitudinal cohorts characterizing natural history with annual examinations and imaging; Michel system validation in athletic populations establishing optimal thresholds; MRI studies testing anatomical predisposition by correlating muscle morphology/nerve variants with symptoms; intervention trials testing preventive strategies; cross-sport comparative studies clarifying biomechanical specificity; and development of objective diagnostic criteria incorporating examination, imaging, and diagnostic injection for definitive prevalence estimation.

## 5. Conclusions

This study demonstrates substantial piriformis syndrome symptom burden (25.4% high scores, 40.3% sitting pain) in high-level hockey players but no significant associations with age, playing duration, competitive level, or status. Within the constraints of this cross-sectional survey design, findings challenge simple cumulative-exposure models and are consistent with anatomical predisposition as a contributing factor, though definitive causal inference is precluded by study design. Symptom pattern homogeneity across demographic subgroups suggests universal symptom screening may be more appropriate than risk-stratified approaches, though implementation should be considered preliminary and investigational pending validation studies demonstrating clinical utility. Any screening program should employ a two-stage methodology (questionnaire followed by clinical examination) rather than relying on survey-based assessment alone. The former player trend warrants longitudinal validation. Future research incorporating clinical examination, diagnostic imaging, and prospective designs is needed to clarify natural history, establish definitive prevalence, and develop evidence-based prevention and treatment strategies for this at-risk population.

## Figures and Tables

**Table 1:** Demographic characteristics and hockey experience of study participants (*n* = 67).

Characteristic	*n* (%)	Statistics
Sex		
Male	58 (86.6)	
Female	9 (13.4)	
Age (years)		
18–20	7 (10.4)	Mean: 25.6 ± 4.0
21–23	16 (23.9)	Median: 27.0
24–26	11 (16.4)	Range: 18–33
27–29	18 (26.9)	IQR: 23–29
30–32	14 (20.9)	
33+	1 (1.5)	
Highest Competition Level		
Junior	2 (3.0)	
Collegiate	55 (82.1)	
Professional	10 (14.9)	
Years of Hockey Experience		
9–12	4 (6.0)	Mean: 17.8 ± 3.7
13–16	14 (20.9)	Median: 18.0
17–20	18 (26.9)	Range: 9–26
21–24	26 (38.8)	IQR: 16–21
25–26	5 (7.5)	
Playing Status		
Currently Active	33 (49.3)	
Retired	34 (50.7)	

**Table 2:** Distribution of piriformis syndrome scores and comparison by playing status (*n* = 67).

Score	Overall *n* (%)	Current Players	Former Players
2	2 (3.0)	1	1
3	8 (11.9)	5	3
4	31 (46.3)	18	13
5	9 (13.4)	4	5
6	7 (10.4)	2	5
7	2 (3.0)	0	2
8	5 (7.5)	2	3
9	1 (1.5)	0	1
10	2 (3.0)	1	1
Summary Statistics			
Mean ± SD	4.8 ± 1.8	4.4 ± 1.7	5.2 ± 1.8
Median	4.0	4.0	4.0
IQR	4–5	4–5	4–6
Range	2–10	2–10	2–10
Binary Classification			
High Score (≥6)	17 (25.4)	5 (15.2)	12 (35.3)
Low Score (<6)	50 (74.6)	28 (84.8)	22 (64.7)

IQR = Interquartile Range; SD = Standard Deviation. Difference between groups: p=0.053 (ANOVA).

## Data Availability

Data available upon reasonable request.

## Abbreviations

The following abbreviations are used in this manuscript:

ACHA	American Collegiate Hockey Association
ANOVA	Analysis of Variance
AUA	Atlantic University Association
CI	Confidence Interval
CT	Computed Tomography
EMG	Electromyography
ESB	Education, Social Science, and Behavioral Research
FAIR	Flexion, Adduction, and Internal Rotation (test)
FHL	Federal Hockey League
HCLK	Hip flexion, adduction, and Lateral Knee (test)
IQR	Interquartile Range
IRB	Institutional Review Board
MRI	Magnetic Resonance Imaging
MSK	Musculoskeletal
NCAA	National Collegiate Athletic Association
NSAIDs	Nonsteroidal Anti-inflammatory Drugs
OMT	Osteopathic Manipulative Treatment
PrP	Platelet-rich Plasma
REDCap	Research Electronic Data Capture
SMS	Short Message Service
SPHL	Southern Professional Hockey League
